# Hypothalamic-pituitary-adrenal stress axis function and the relationship with chronic widespread pain and its antecedents

**DOI:** 10.1186/ar1772

**Published:** 2005-06-17

**Authors:** John McBeth, Yee H Chiu, Alan J Silman, David Ray, Richard Morriss, Chris Dickens, Anindya Gupta, Gary J Macfarlane

**Affiliations:** 1 Arthritis Research Campaign (ARC) Epidemiology Unit, School of Epidemiology and Health Sciences, University of Manchester, Manchester, United Kingdom; 2 Unit of Chronic Disease Epidemiology, School of Epidemiology and Health Sciences, University of Manchester, Manchester, United Kingdom; 3 Endocrine Sciences Research Group, University of Manchester, Manchester, United Kingdom; 4 University Department of Psychiatry, Royal Liverpool University Hospital, Liverpool, United Kingdom; 5 Department of Psychiatry, University of Manchester, United Kingdom

## Abstract

In clinic studies, altered hypothalamic-pituitary-adrenal (HPA) axis function has been associated with fibromyalgia, a syndrome characterised by chronic widespread body pain. These results may be explained by the associated high rates of psychological distress and somatisation. We address the hypothesis that the latter, rather than the pain, might explain the HPA results. A population study ascertained pain and psychological status in subjects aged 25 to 65 years. Random samples were selected from the following three groups: satisfying criteria for chronic widespread pain; free of chronic widespread pain but with strong evidence of somatisation ('at risk'); and a reference group. HPA axis function was assessed from measuring early morning and evening salivary cortisol levels, and serum cortisol after physical (pain pressure threshold exam) and chemical (overnight 0.25 mg dexamethasone suppression test) stressors. The relationship between HPA function with pain and the various psychosocial scales assessed was modelled using appropriate regression analyses, adjusted for age and gender. In all 131 persons with chronic widespread pain (participation rate 74%), 267 'at risk' (58%) and 56 controls (70%) were studied. Those in the chronic widespread pain and 'at risk' groups were, respectively, 3.1 (95% CI (1.3, 7.3)) and 1.8 (0.8, 4.0) times more likely to have a saliva cortisol score in the lowest third. None of the psychosocial factors measured were, however, associated with saliva cortisol scores. Further, those in the chronic widespread pain (1.9 (0.8, 4.7)) and 'at risk' (1.6 (0.7, 3.6)) groups were also more likely to have the highest serum cortisol scores. High post-stress serum cortisol was related to high levels of psychological distress (p = 0.05, 95% CI (0.02, 0.08)). After adjusting for levels of psychological distress, the association between chronic widespread pain and post-stress cortisol scores remained, albeit slightly attenuated. This is the first population study to demonstrate that those with established, and those psychologically at risk of, chronic widespread pain demonstrate abnormalities of HPA axis function, which are more marked in the former group. Although some aspects of the altered function are related to the psychosocial factors measured, we conclude that the occurrence of HPA abnormality in persons with chronic widespread pain is not fully explained by the accompanying psychological stress.

## Introduction

Fibromyalgia is a common syndrome of which chronic widespread body pain is the cardinal feature [1]. We have previously shown in a community-based prospective cohort study, the Altrincham Pain Study, that psychosocial factors, including reporting other non-pain somatic symptoms, aspects of illness behaviour and high levels of psychological distress and fatigue, were the strongest predictors of the onset of chronic widespread pain [2]. The biological processes through which these psychosocial risk factors may lead to pain, however, are unknown.

One hypothesis, although the link is debated [3,4], relates to altered function of the hypothalamic-pituitary-adrenal (HPA) stress axis [5]. Specifically, clinic patients with fibromyalgia have been reported to have hypoactive HPA axis function, with several studies showing reduced basal plasma cortisol, decreased 24-h urinary free cortisol excretion [6-9] and a blunted response to dynamic testing [9-11], Further, pain sensitivity is inversely related to the level of activation of the HPA system's response to stress. The hypoactive HPA axis response of fibromyalgia patients is more marked when compared to that of other chronic pain patients with less widespread symptoms, including those with rheumatoid arthritis [7] and non-inflammatory low back pain [9]. These findings are equivocal, however, with some investigators reporting that patients with fibromyalgia display hyperactive HPA responses [7,8,12,13].

The nature of the relationship between chronic widespread pain and HPA axis function is therefore unclear and remains to be clarified. The studies discussed above were conducted on small numbers of patients, which may have had an impact on the accuracy of the findings. More importantly, subjects with chronic widespread pain, when compared to pain free controls, have increased rates of other symptoms, including high levels of psychological distress [14], notably depressive disorders [15], which are associated with altered HPA axis function. Indeed, mood disorders are even more apparent in those who consult with pain [16]. We have conducted a study to investigate the independent effects of pain and psychological distress in relation to the HPA response.

## Methods

### Design

We conducted a cross-sectional population-based study in which groups of subjects were identified based on their pain and psychological status and whose HPA axis function was assessed. Subjects completed a questionnaire that enquired about aspects of psychological status together with a history of current pain. Those who agreed to further contact by the study team were asked if they would be willing to further participate in a detailed assessment that included measures of HPA axis function. Of those persons agreeing, several subjects had to be excluded (Additional file 1), as accurate HPA assessment in them would not have been possible. Of those remaining, random samples of subjects stratified by pain and psychological distress status were subsequently invited to attend.

### Study sampling frame

Subjects were recruited from the population registers of individuals eligible to receive care from three primary care physicians in the United Kingdom. In total 2,312 subjects aged between 25 and 65 years completed the questionnaire, were eligible to participate further, and formed the sampling frame for the study.

### Ascertainment of pain and psychological status

All subjects completed a questionnaire that included a blank body manikin on which subjects were asked to indicate the site of any pain lasting at least 24 h or more in the past month. The questionnaire also included the following items to ascertain psychological status, including several scales we have previously demonstrated to be associated with the development and persistence of chronic widespread pain [2,17].

#### The General Health Questionnaire

The General Health Questionnaire (GHQ) [18] is a 12-item questionnaire that was originally developed as a screening instrument for mental disorder in the community. It has been widely used in population-based studies as a measure of psychological distress. Each item response scores 0 or 1. Total scores range from 0 to 12, with higher scores indicating higher levels of psychological distress.

#### The Illness Attitude Scales

The nine scales of the Illness Attitude Scales [19] individually measure worries about health, concern about pain, hypochondriacal beliefs, health habits, bodily preoccupation, fear of dying, disease phobia, treatment experience and effect of symptoms. Based on the scores generated, two subscales have been identified, Health Anxiety and Illness Behaviour [21].

#### The Hospital Anxiety and Depression scales

The Hospital Anxiety and Depression (HAD) [21] was originally developed for use in patients with physical illnesses but is widely used in population-based studies. The 14 items, each being scored on a 0 to 3 scale, measure degrees of anxiety (seven items) and depression (seven items). The two subscale scores thus range from 0 to 21, with higher scores indicating an increased likelihood of an anxiety or depressive disorder being present.

#### The List of Threatening Experiences

The List of Threatening Experiences [22] is a 12-event inventory initially modified by Bebbington and colleagues [22] from a 67 life-events inventory introduced by Tennant and Andrews [23]. The categories ask about recent adverse experiences of personal relationships, employment, illness, and financial and legal issues in the last six months.

#### The Sleep Problem Scale

The Sleep Problem Scale [24] contains four items that examine recent problems with sleep. Responses are scored in a range of 0 to 5, giving a total score of between 0 and 20. Higher scores indicate increased sleep disturbance.

### Classification of study groups

To examine the study hypotheses three groups of subjects, classified according to their pain reports and psychological status, were selected to participate in an assessment of their HPA axis function. Informed consent to participate in the study was sought from all subjects.

#### Chronic widespread pain group

This group of subjects comprised those with chronic widespread pain, classified according to the American College of Rheumatology criteria [1]. This group of subjects included all those who satisfied those criteria, irrespective of their tender point count.

#### 'At risk' group

This group of subjects comprised those without chronic widespread pain but who, based on their psychological status (reporting three or more symptoms on the Somatic Symptom Checklist and scoring 5 or more on the Illness Behaviour Scale) and as demonstrated in our previous work [2], were at risk of its future development. This group of subjects have many features in common with the Chronic widespread pain group, though were free of chronic widespread pain.

#### Reference group

A group of individuals who neither had current chronic widespread pain nor showed evidence of being psychologically distressed were classified as a reference group.

### Assessment of HPA axis function

Methods were selected appropriate for undertaking in large samples of community dwelling subjects, though there are no standardised methods of assessing HPA function in such studies. We therefore decided to take a broad approach and used four measures of axis function: two that assessed HPA tone using salivary cortisol levels and two that assessed HPA response to different stimuli based on serum levels. Salivary cortisol affords a convenient method of assessment that provides a valid and reliable correlate of serum/plasma free diurnal cortisol levels [25,26]. This method is easy to administer, especially in studies recruiting large numbers of subjects, and minimises disruption to participants' lives.

Subjects collected the salivary cortisol levels in the evening (at 10 pm) and the following morning (between 8 am and 9 am). Serum cortisol levels were measured after an overnight 0.25 mg dexamethasone suppression test and a physical (pain pressure threshold examination) stressor (see Additional file 2 for sample collection schedule).

### Hormone assays

Plasma cortisol was determined using the Chiron Diagnostic ACS:180 analyser (Bayer HealthCare LLC Diagnostic Division, New York, NY, USA). The assay is a competitive chemiluminescent immunoassay. Cortisol in the sample competes with cortisol labelled with acridinium ester for a limited amount of polyclonal rabbit anti-cortisol antibody coupled to paramagnetic particles. The cortisol concentration in the sample is inversely proportional to the relative light units detected in the ACS:180 system.

A modified version of the radioimmunoassay method used for serum cortisol was employed for assaying salivary cortisol levels. This method depends on competition between cortisol present in sample or standard and ^125^I-labelled cortisol for a limited number of binding sites on rabbit ant-cortisol antibody. Separation of the antibody-bound fraction is effected by incubation with donkey anti-rabbit antibody coated to cellulose particles followed by centrifugation and decantation of the supernatant. The pellet is then counted and the amount of tracer bound is inversely proportional to the concentration of cortisol present. Before assay, the salivary sample was centrifuged for 5 minutes at 2500 revolutions per minute. The supernatant was then removed for assay.

Ethical approval was sought and granted from the appropriate Local Research Ethics Committees. The Arthritis Research Campaign, Chesterfield, England, funded the study.

### Statistical analysis

We compared the raw cortisol data for each of the four assessment methods between the three groups of participants. The individual methods used to assess HPA function were inevitably prone to random error owing to difficulties in standardising collection procedures in the home environment. We therefore examined whether an approach that combined the four sources of cortisol data would be more informative. To do this we used a principal component analysis (PCA), a statistical technique that reduces the complexity of a number of inter-correlated variables by reduction of the observed data to a smaller number of uncorrelated variables called the principal components. The individual values of the observed variables contribute ('load on') to the resulting component scores, which are calculated for each subject. PCA analysis produces eigenvalues, a measure of the variance that is explained by the individual components. Importantly, PCA does not require prior assumptions about individual variable interactions. Conventionally, eigenvalues over 1 are said to explain more of the variance than the raw data alone. For analysis, the resulting principal component scores were categorized into tertiles based upon the distribution of scores with the highest or lowest tertile classified as the referent category. The relationship with those persons 'at risk' of, or having chronic widespread pain compared to the reference group and component scores was expressed as Odds Ratios (ORs) with 95% confidence intervals (CI). The relationship between component scores and psychosocial scales was modelled using linear regression. Due to high correlation coefficients (r > 0.4), we removed the HAD anxiety (correlated with the GHQ and Health Anxiety scale) and HAD depression (correlated with the GHQ and Sleep scale) subscales from this analysis. All analyses were adjusted for the potential confounding effects of age and gender.

## Results

### Response and participation rates

**Figure 1:**
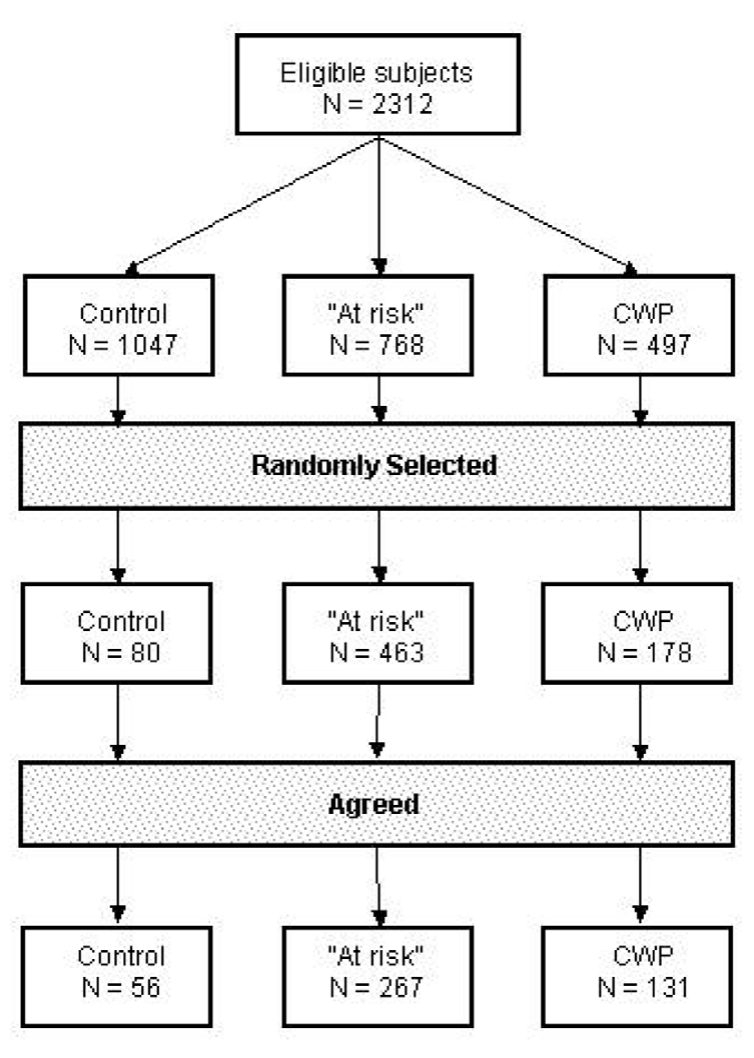
Flow of study subjects. CWP, chronic widespread pain.

Of the 2,312 eligible subjects, 497 (13%) had chronic widespread pain, 768 (21%) were free of chronic widespread pain but at risk of its future development, and 1047 (28%) were in the reference group (Fig. 1). Random samples of 178, 463 and 80 subjects from each of these three groups, respectively, were telephoned and invited to the assessment of HPA axis function of which 131 (74%), 267 (58%) and 56 (70%), respectively, agreed to participate. An analysis of the distribution of the questionnaire variables between those subjects within each of these three sample groups who did and did not agree to participate was undertaken and showed no evidence of non-participation bias (data not shown). A total of 429 subjects (125 with chronic widespread pain, 254 at risk and 50 controls) provided all four measures of HPA axis function and were included in the current analysis. The cortisol data were examined for outliers, with values more than 4 standard deviations from the mean being eliminated. This procedure resulted in four (0.9%) observations being removed (one person with chronic widespread pain and three 'at risk'), leaving a total of 425 subjects for analysis. Of those who participated, there were no significant differences between the three groups in age or gender (Table 1); however, persons 'at risk' and those with chronic widespread pain, as expected, had significantly higher scores on all of the psychosocial scales when compared to the reference group.

### Results from the principal components analysis

**Figure 2:**
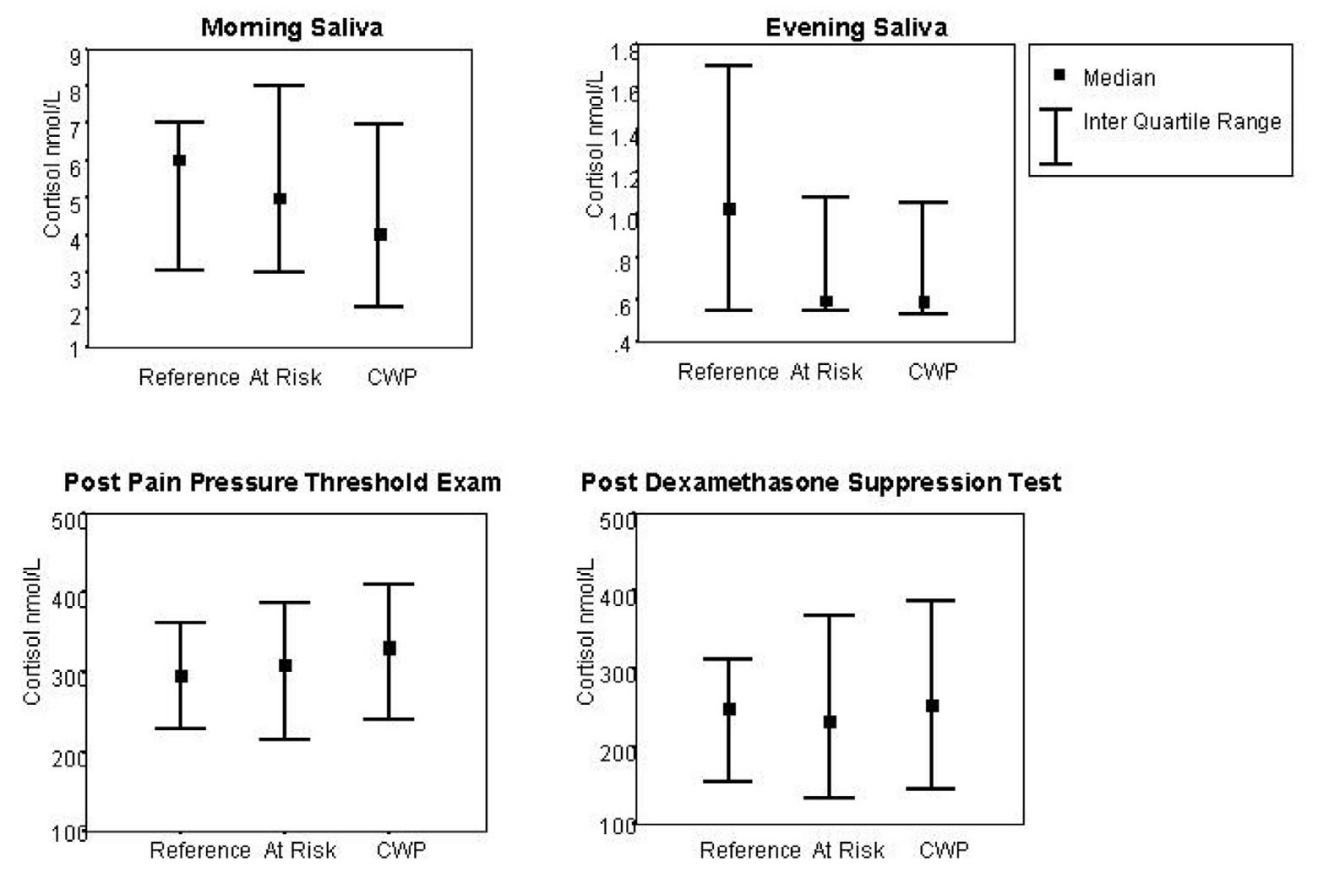
Distribution (median, inter-quartile range) of cortisol levels by study group. CWP, chronic widespread pain.

An examination of the raw data revealed a tendency towards lower saliva cortisol levels and higher post-stress levels in both those at risk and those with established chronic widespread pain (Fig. 2, Table 2). The PCA yielded four composite cortisol scores (Table 3), three of which had eigenvalues above or approaching 1. These three components were interpreted as: a 'saliva cortisol' score that was composed of the evening and morning cortisol values; a 'post-stress cortisol' score that was composed of the post-dexamethasone and post-physical exam cortisol scores; and a 'post-stress difference' cortisol score that was composed of the difference between post-dexamethasone and post-physical exam scores. These three components were used in all further analyses.

### Principal components of HPA function and pain

**Figure 3:**
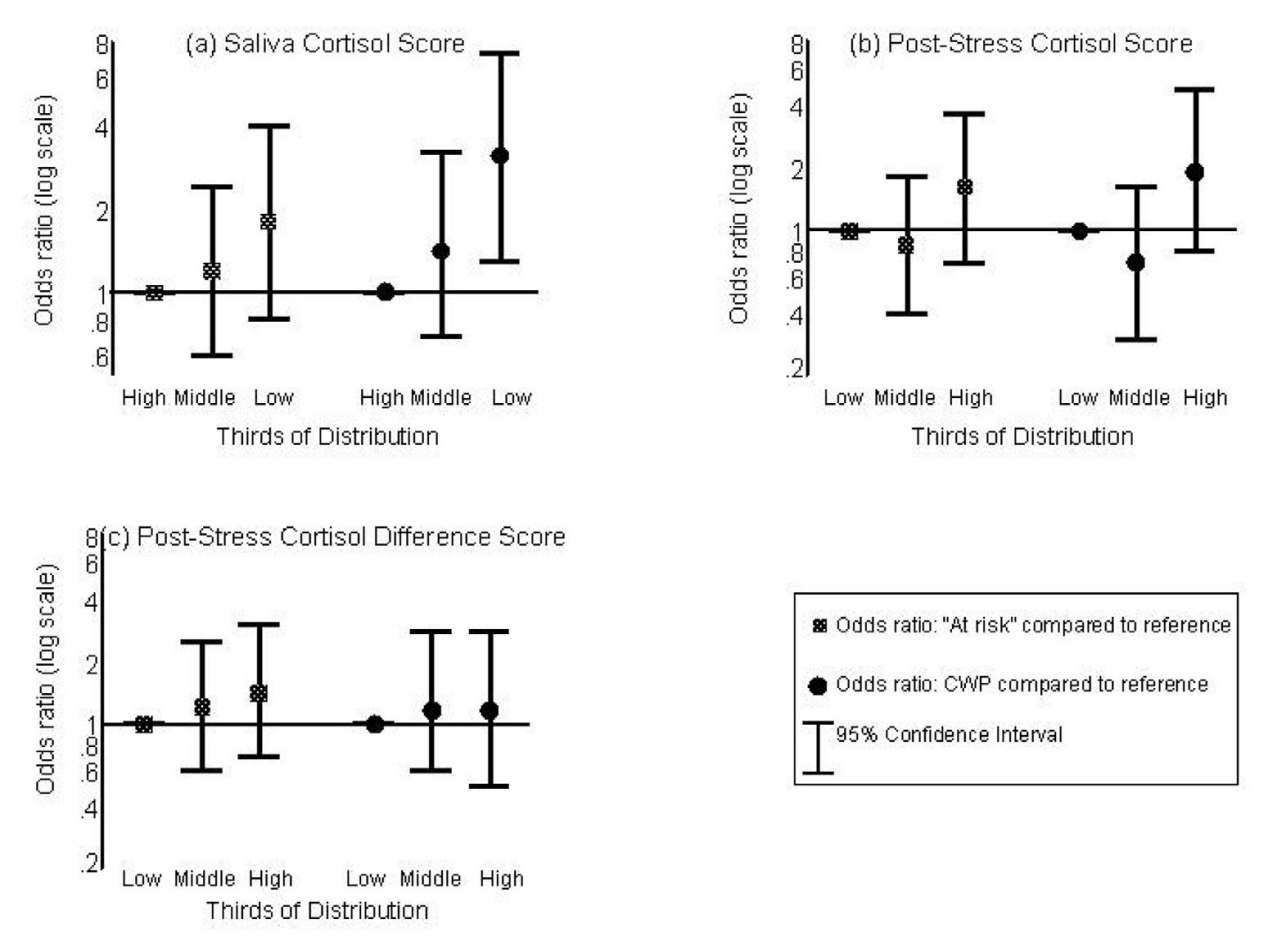
Results of logistic regression models of the association between study groups and cortisol levels. All analyses were adjusted for age and gender. CWP, chronic widespread pain.

Compared to the reference group, those persons at risk of future chronic widespread pain were 1.8 times more likely to have a saliva cortisol score in the lowest third, while those with chronic widespread pain were three times more likely (Fig. 3a). Both subjects 'at risk' and those with chronic widespread pain had higher, though non-significant, post-stress cortisol scores, being 1.6 and 1.9-times more likely, respectively, to have the highest post-stress serum cortisol scores when compared to the reference group (Fig. 3b). Neither being at risk of nor having chronic widespread pain was associated with post-stress difference scores (Fig. 3c).

The relationships between HPA axis function and being in the 'at risk' or chronic widespread pain groups compared to the reference group were strong, although, as discussed above, may have been explained by the presence of psychosocial factors. We therefore examined the relationship between psychosocial factors and HPA axis function. A linear regression analysis revealed that none of the psychosocial factors measured were associated with saliva cortisol scores (Table 4). Higher levels of psychological distress, however, were associated with higher post-stress serum cortisol scores (β = 0.05, 95% CI (0.02, 0.08)) and, although not statistically significant, recent life events also showed a positive association. After adjusting for the effects of these variables, the relationship between being at risk of (OR = 1.3, 95% CI (0.5, 3.0)) or having chronic widespread pain (OR = 1.8, 95% CI (0.7, 4.6)) and post-stress cortisol scores was further attenuated.

## Discussion

Clinic based studies have suggested that persons with chronic widespread pain display altered HPA axis function. Due to small numbers of subjects and the presence of psychological factors that may confound the association, however, the true relationship remained unclear. In this, the first community-based study, we have demonstrated that chronic widespread pain was associated with altered HPA axis function. Specifically, the presence of chronic widespread pain was associated with lower levels of salivary cortisol and higher levels of post-stressor serum cortisol. Interestingly, one would expect, under normal circumstances, an exaggerated HPA response to the pain threshold examination and a blunted response following the dexamethasone suppression test as these two measures, although 'stress response' measures, are testing different functions of the HPA axis. Our data indicate that both a high post-pain threshold level of cortisol and a failure to suppress after the dexamethasone suppression test were associated with having chronic widespread pain. High levels of psychological distress did not explain the relationship with salivary cortisol levels, although the relationship with high levels of post-stress serum cortisol was explained to some extent by the presence of psychological distress.

In considering these results it is useful to highlight some of the methodological issues that may have had an impact on our findings. First, a number of those subjects who were invited to participate refused to do so. On analysis of the questionnaire data within the groups selected to participate we found that there were no significant differences in the age, gender or psychological status between those who did and did not agree. Second, and more importantly, the measures used to assess HPA function were not as rigorous as those used in laboratory based studies, although they were more wide-ranging. In addition, since subjects were relied upon to take the dexamethasone tablets and collect the salivary samples in the absence of a member of the study team, we did not have as much control over the sample collection. These factors are likely to have introduced 'noise' into the data collected and, because it is likely that any deviance from the study schedule was random across the study groups, such 'noise' would act to make it harder to find an association. That being the case, it is also likely that any association we have reported is an underestimate of the true association.

There are no comparable community-based studies with which to compare our findings, although clinic based studies of fibromyalgia patients have reported a range of HPA axis disruptions. Thus, Crofford and colleagues [8] reported that fibromyalgia clinic patients had low 24-h urinary free cortisol levels and low levels of cortisol in response to challenge with ovine corticotropin-releasing hormone when compared to age- and sex-matched pain free controls. Griep and colleagues [9] examined the HPA axis function in a group of 40 patients with fibromyalgia, 28 with non-inflammatory low back pain and 14 pain free controls. Compared to the pain free control subjects, those with fibromyalgia displayed mild hypocortisolemia and significantly lower levels of 24-h urinary free cortisol. Subjects with low back pain showed similar perturbations but to a lesser extent than those observed in the group of fibromyalgia patients.

## Conclusion

We have previously shown that psychological status is a strong predictor of the onset of chronic widespread pain [2], although the biological mechanism through which such factors may lead to pain was unclear. The altered HPA function evident in subjects in the present study is one possible mechanism. We propose that the impaired HPA axis tone as shown by the low salivary cortisol measurements indicates a failure to mount an adequate stress response to psychological insult, and that this failure predisposes to the development of chronic widespread pain. However, this model can only be examined in a prospective study. To that end we are following-up those persons in the current study who were 'at risk' of the future development of symptoms to determine whether, among that group of subjects who are psychologically distressed, altered HPA function predicts symptom onset.

## Abbreviations

CI = confidence interval; CWP = chronic widespread pain; GHQ = General Health Questionnaire; HAD = Hospital Anxiety and Depression; HPA = hypothalamic-pituitary-adrenal; IQR = inter-quartile range; OR = odds ratio; PCA = principal component analysis.

## Competing interests

The authors declare that they have no competing interests.

## Authors' contributions

JM, DR, AJS, RM, CD and GJM were responsible for study development and design. All authors were responsible for study conduct and manuscript revisions. JM conducted data analysis and prepared the manuscript. All authors have access to all data in the study and hold final responsibility for the decision to submit for publication.

## Supplementary Material

Additional File 1 A word file showing phase 2 inclusion and exclusion criteria Click here for file

Additional File 2 A word file showing the schedule for endocrine tests Click here for file

## Figures and Tables

**Table 1:** Distribution of age, gender and scale score by pain group

	Controls (N = 50)		At risk (N = 251)		p-value^a^	CWP (N = 124)		p-value^b^
	n	%	n	%		n	%	

Age group								
25–43	16	32	91	36		40	30	
44–54	18	36	78	31		46	37	
55–65	16	32	82	33	0.85	38	33	0.78
Gender								
Female	30	60	165	66	0.44	74	60	0.97
								

	Median	IQR	Median	IQR		Median	IQR	

Sleep	3	0–5	6	3–11	0.00	9	5–13	0.00
GHQ	0	1–1.5	1	0–5	0.00	1	0–5	0.00
Somatic symptoms	0	0-0	1	1–2	0.00	1	0–2	0.00
Health anxiety	8	4–11	11	6–15	0.00	9	6–15	0.03
Illness behaviour	2	1–4	6	5–9	0.00	6	4–10	0.00
HAD: anxiety	4	2–7	6	4–9	0.00	7	4–10	0.00
HAD: depression	1	1–4	3	1–6	0.00	5	2–7	0.00
Life events	0	0–1	1	0–2	0.01	1	0–2	0.00

^a^At risk compared to controls. ^b^CWP compared to controls. All p-values were by Mann-Whitney U test except those for sex, which were by chi-square test. CWP, chronic widespread pain; GHQ, General Health Questionnaire; HAD, Hospital Anxiety and Depression scale; IQR, inter-quartile range.

**Table 2:** Distribution of cortisol levels by pain group

Cortisol measures (nmol/l)	Controls (N = 50)		At risk (N = 251)		CWP (N = 124)	
	Median	IQR	Median	IQR	Median	IQR

Saliva						
Morning (nmol/l)	5	3–7	5	3–8	4	2–7
Evening (nmol/l)	1	0.5–1	0.5	0.5–1	0.5	0.5–1
						
Serum						
Post-physical exam (nmol/l)	295	229–362	310	215–387	331	240–410
Post-suppression test (nmol/l)	249	154–313	232	133–368	253	144–388

CWP, chronic widespread pain; IQR, inter-quartile range.

**Table 3:** Results of principal components analysis

	Component loadings			
	1	2	3	4

Post exam serum	-0.14	0.68	0.71	0.04
Post dexamethasone serum	-0.08	0.71	-0.70	0.04
Saliva morning	0.69	0.15	0.03	-0.70
Saliva evening	0.70	0.07	0.03	0.71
Eigenvalue	1.89	1.18	0.81	0.13
Variance explained (%)	47	29	20	3

**Table 4:** Association between psychosocial scales and cortisol levels

Scale	Component					
	Saliva		Post-stress		Post-stress difference	

	β	95% CI	β	95% CI	β	95% CI

Sleep	-0.003	-0.006, 0.001	0.009	-0.011, 0.028	0.005	-0.011, 0.021
GHQ	-0.004	-0.010, 0.002	0.052^a^	0.021^a^, 0.083^a^	0.008	-0.017, 0.034
Somatic symptoms	0.023	-0.002, 0.048	-0.097	-0.231, 0.037	0.086	-0.022, 0.193
Illness behaviour	0.004	-0.002, 0.010	-0.011	-0.040, 0.019	-0.026	-0.050, -0.002
Health anxiety	-0.000	-0.003, 0.003	0.012	-0.003, 0.027	0.013	0.000, 0.025
Recent life events	0.001	-0.014, 0.016	0.040	-0.039, 0.119	0.024	-0.042, 0.090

Analyses have been adjusted for age and gender. ^a^Statistically significant results. CI, confidence interval; GHQ, General Health Questionnaire.

## References

[B1] Wolfe F, Smythe HA, Yunus MB, Bennett RM, Bombardier C, Goldenberg DL, Tugwell P, Campbell SM, Abeles M, Clark P (1990). The American College of Rheumatology 1990 criteria for the classification of fibromyalgia. Report of the Multicenter Criteria Committee. Arthritis Rheum.

[B2] McBeth J, Macfarlane GJ, Benjamin S, Silman AJ (2001). Features of somatization predict the onset of chronic widespread pain: results of a large population-based study. Arthritis Rheum.

[B3] Tsigos C, Chrousos GP (2002). Hypothalamic-pituitary-adrenal axis, neuroendocrine factors and stress. J Psychosom Res.

[B4] Geenen R, Jacobs JW, Bijlsma JW (2002). Evaluation and management of endocrine dysfunction in fibromyalgia. Rheum Dis Clin North Am.

[B5] Okifuji A, Turk DC (2002). Stress and psychophysiological dysregulation in patients with fibromyalgia syndrome. Appl Psychophysiol Biofeedback.

[B6] Clauw DJ, Chrousos GP (1997). Chronic pain and fatigue syndromes: overlapping clinical and neuroendocrine features and potential pathogenic mechanisms. Neuroimmunomodulation.

[B7] McCain GA, Tilbe KS (1989). Diurnal hormone variation in fibromyalgia syndrome: a comparison with rheumatoid arthritis. J Rheumatol Suppl.

[B8] Crofford LJ, Pillemer SR, Kalogeras KT, Cash JM, Michelson D, Kling MA, Sternberg EM, Gold PW, Chrousos GP, Wilder RL (1994). Hypothalamic-pituitary-adrenal axis perturbations in patients with fibromyalgia. Arthritis Rheum.

[B9] Griep EN, Boersma JW, Lentjes EG, Prins AP, van der Korst JK, de Kloet ER (1998). Function of the hypothalamic-pituitary-adrenal axis in patients with fibromyalgia and low back pain. J Rheumatol.

[B10] Riedel W, Layka H, Neeck G (1998). Secretory pattern of GH, TSH, thyroid hormones, ACTH, cortisol, FSH, and LH in patients with fibromyalgia syndrome following systemic injection of the relevant hypothalamic-releasing hormones. Z Rheumatol.

[B11] Griep EN, Boersma JW, de Kloet ER (1993). Altered reactivity of the hypothalamic-pituitary-adrenal axis in the primary fibromyalgia syndrome. J Rheumatol.

[B12] Hudson JI, Pliner LF, Hudson MS, Goldenberg DL, Melby JC (1984). The dexamethasone suppression test in fibrositis. Biol Psychiatry.

[B13] Ferraccioli G, Cavalieri F, Salaffi F, Fontana S, Scita F, Nolli M, Maestri D (1990). Neuroendocrinologic findings in primary fibromyalgia (soft tissue chronic pain syndrome) and in other chronic rheumatic conditions (rheumatoid arthritis, low back pain). J Rheumatol.

[B14] Hunt IM, Silman AJ, Benjamin S, McBeth J, Macfarlane GJ (1999). The prevalence and associated features of chronic widespread pain in the community using the 'Manchester' definition of chronic widespread pain. Rheumatology (Oxford).

[B15] Benjamin S, Morris S, McBeth J, Macfarlane GJ, Silman AJ (2000). The association between chronic widespread pain and mental disorder: a population-based study. Arthritis Rheum.

[B16] Macfarlane GJ, Morris S, Hunt IM, Benjamin S, McBeth J, Papageorgiou AC, Silman AJ (1999). Chronic widespread pain in the community: the influence of psychological symptoms and mental disorder on healthcare seeking behavior. J Rheumatol.

[B17] McBeth J, Macfarlane GJ, Hunt IM, Silman AJ (2001). Risk factors for persistent chronic widespread pain: a community-based study. Rheumatology (Oxford).

[B18] Goldberg DP, Williams P (1988). A User's Guide to the General Health Questionnaire.

[B19] Kellner R (1994). Psychosomatic syndromes, somatization and somatoform disorders. Psychother Psychosom.

[B20] Speckens AE, Spinhoven P, Sloekers PP, Bolk JH, Van Hemert AM (1996). A validation study of the Whitely Index, the Illness Attitude Scales, and the Somatosensory Amplification Scale in general medical and general practice patients. J Psychosom Res.

[B21] Zigmond AS, Snaith RP (1983). The hospital anxiety and depression scale. Acta Psychiatr Scand.

[B22] Brugha T, Bebbington P, Tennant C, Hurry J (1985). The List of Threatening Experiences: a subset of 12 life event categories with considerable long-term contextual threat. Psychol Med.

[B23] Tennant C, Andrews G (1976). A scale to measure the stress of life events. Aust N Z J Psychiatry.

[B24] Jenkins CD, Stanton BA, Niemcryk SJ, Rose RM (1988). A scale for the estimation of sleep problems in clinical research. J Clin Epidemiol.

[B25] Kirschbaum C, Hellhammer DH (1994). Salivary cortisol in psychoneuroendocrine research: recent developments and applications. Psychoneuroendocrinology.

[B26] Vedhara K, Miles J, Bennett P, Plummer S, Tallon D, Brooks E, Gale L, Munnoch K, Schreiber-Kounine C, Fowler C (2003). An investigation into the relationship between salivary cortisol, stress, anxiety and depression. Biol Psychol.

