# Supplementary material for: Hypothalamic-pituitary-adrenal stress axis function and the relationship with chronic widespread pain and its antecedents
Source: Arthritis Res Ther. 2005 Jun 17;7(5):R992–R1000. doi: 10.1186/ar1772 (PMC1257426; doi:10.1186/ar1772)
Supplement: Additional File 1 — A word file showing phase 2 inclusion and exclusion criteria [file ar1772-S1.doc]

# Additional file 1

Phase 2 inclusion and exclusion criteria

Inclusion

To be eligible to enter phase 2 of the study, subjects had to have fully completed all aspects of the first phase questionnaire and consented to further contact by the study team.

Exclusion

Subjects were excluded if they reported any of the following:

- insulin dependant diabetes mellitus
- epilepsy
- acute or serious medical condition (e.g. malignancy, stroke, myocardial infarction)
- anticonvulsant therapy
- pregnancy
- oral contraceptive use (oestrogens and/or progesterone)
- hormone replacement therapy use
- steroid use (including inhaled or nasal delivery preparations in the last six months)
- current or recent participation in another study or clinical trial and under follow up for such
