# Supplementary material for: Hypothalamic-pituitary-adrenal stress axis function and the relationship with chronic widespread pain and its antecedents
Source: Arthritis Res Ther. 2005 Jun 17;7(5):R992–R1000. doi: 10.1186/ar1772 (PMC1257426; doi:10.1186/ar1772)
Supplement: Additional File 2 — A word file showing the schedule for endocrine tests [file ar1772-S2.doc]

# Additional file 2

Schedule for endocrine tests

Methods were selected appropriate for undertaking in large samples of community dwelling subjects, though there are no standardised methods of assessing HPA function in such studies. We therefore decided to take a broad approach and used four measures of axis function: one that assessed serum cortisol levels in response to an acute stressor, a pain-threshold examination; one that assessed serum cortisol levels in response to a low dose dexamethasone suppression test; and two that assessed diurnal HPA tone using salivary cortisol levels. All tests were carried out and samples collected in subject’s homes or at their local general practitioners office. The rationale for using these tests and sample collection methodologies are described below:

1. Post pain-threshold examination serum cortisol levels

Although there is no precedent for collection and interpretation of cortisol levels following a pain-threshold examination, we hypothesised that serum cortisol levels collected in this manner would represent HPA axis response to an acute stressor. Pain threshold was measured using a Fischer pressure threshold meter [1] at eight bilateral body sites: anterior border of the tibia; medial fat pad of the knee; gluteus maximus; medial supraspinatus; deltoid; lateral epicondyle; dorsum of the forearm; and the trapezius. The pressure threshold meter is a force gauge calibrated in kg/cm^2^, with a range of 0 to 20 kg. Pressure was applied at the predefined sites at an increasing rate of 1kg/sec. Subjects were asked to indicate the point a pressure sensation became a painful sensation by saying “now”, at which point the pressure that had been applied was read from the meter and recorded. The examination lasted a total of 10 minutes and a blood sample was taken 30 minutes from the start of the procedure.

1. Low dose overnight dexamethasone suppression test

Lower levels of adrenocorticotrophin hormone (ACTH) are released by the HPA axis in response to increased circulating blood levels of glucocorticoids, resulting in lower levels of cortisol being produced by the adrenal glad. The integrity of this feedback mechanism can be tested by administering an evening low dose of dexamethasone, a potent glucocorticoid, and judging morning ACTH secretion by analysis of plasma cortisol levels [2]. The morning level of plasma cortisol in normal subjects will be <140 nmol/l [2]. In the current study, we used 0.25 mg of dexamethasone [3,4]. Dexamethasone was administered at 10 pm [5] and a blood sample was taken by a research nurse at 8 am.

1. Diurnal salivary cortisol levels

Salivary cortisol affords a convenient method of assessment that provides a valid and reliable correlate of serum/plasma free diurnal cortisol levels [6,7]. Salivary cortisol has several advantages over blood cortisol analysis being non-invasive and therefore stress-free sampling, is easy to administer, especially in studies recruiting large numbers of subjects, and minimises disruption to participants’ lives. Subjects were supplied with a standardised protocol detailing the sample collection schedule. Saliva samples were obtained by soaking a swab for 90 seconds in the evening (at 10 pm) and one swab the following morning (between 8 and 9 am). Following saliva collection the swabs were placed in the freezer to be collected by a research nurse.

- Smokers will need to abstain for at least one hour prior to samples being taken (nicotine elevates cortisol levels); likewise any products containing nicotine such as gum or patches will need to be avoided. Subjects will also need to avoid drinking alcohol on the day of the test.
- We would aim for women to have their investigations between day 1 and 10 of their menstrual cycle. Menstrual cycle phase may effect hormonal levels and test results.
- Women should remove lipstick prior to producing saliva for collection (interaction with reagents used in cortisol assay).
- Subjects should not brush their teeth or eat until after they produce their saliva samples.

###### Day 1

1. 9 am saliva sample

2. 10 pm saliva sample

3. Pain pressure threshold examination, with plasma cortisol level

4. 10 pm 0.25mg dexamethasone

5. Fast from this point till morning sample of cortisol taken

Day 2

1. 9 am plasma cortisol level.

2. Subjects may eat and drink normally once samples taken
